# Do CVID patients on SCIG have more autoimmune (thrombo)cytopenic events than CVID patients on IVIG?

**DOI:** 10.3389/fimmu.2025.1708813

**Published:** 2025-11-27

**Authors:** Nadezhda Camacho-Ordonez, Aleksandra Hirsch, Luiza Campos, Sigune Goldacker, Siobhan O. Burns, Fernando Moreira, Klaus Warnatz, Bodo Grimbacher

**Affiliations:** 1 Institute for Immunodeficiency, Center for Chronic Immunodeficiency, University Medical Center Freiburg, Freiburg, Germany; 2 Department of Rheumatology and Clinical Immunology, University Medical Center Freiburg, Freiburg, Germany; 3 Department of Immunology, Royal Free London NHS Foundation Trust, London, United Kingdom; 4 Institute of Immunity and Transplantation, University College London, London, United Kingdom; 5 Center for Integrative Biological Signaling Studies (CIBSS), University of Freiburg, Freiburg, Germany; 6 Resolving Infection Susceptibility (RESIST) - Cluster of Excellence 2155 to Hannover Medical School, Satellite Center Freiburg, Freiburg, Germany; 7 German Center for Infection Research (DZFI), Satellite Center Freiburg, Freiburg, Germany

**Keywords:** autoimmune thrombocytopenia, intravenous or subcutaneous immunoglobulin, common variable immunodeficiency, autoimmunity, cytopenia

## Abstract

Autoimmune thrombocytopenia (AITP) is frequent in patients diagnosed with common variable immunodeficiency (CVID). High dose intravenous immunoglobulin treatment (IVIG) has conventionally been a cornerstone of the initial therapy for AITP. This study aimed to assess the safety and effectiveness of subcutaneous immunoglobulin (SCIG) compared to IVIG in preventing AITP in CVID patients. This prospective observational study enrolled 47 adult CVID patients concurrently diagnosed with AITP. Of the participants, 27 (57%) were treated with SCIG, while 20 (43%) received IVIG. AITP episodes were defined as platelet counts <50,000/µl with bleeding or <20,000/µl with or without bleeding, followed over a 64-month period. Among the 47 patients included, 12 (25.5%) experienced AITP episodes, with seven using SCIG and five using IVIG. No significant difference was observed in AITP occurrence between the two treatment groups (p-value=0.99). Neither splenomegaly nor the use of immunosuppressive therapies showed a correlation to the AITP bouts. Maintaining IgG trough levels above 7g/l arose as a key factor for preventing AITP in both treatment modalities. In conclusion, both SCIG and IVIG demonstrated comparable efficacy in the prevention of AITP in CVID patients. This study highlights the importance of monitoring IgG levels in the management of CVID patients with AITP.

## Introduction

Common variable immunodeficiency (CVID) is a complex and clinically heterogeneous primary immunodeficiency disorder (1). A hallmark feature of CVID is hypogammaglobulinemia, resulting in recurrent and severe respiratory infections due to impaired antibody production (2). Beyond its infectious consequences, CVID has a broad spectrum of autoimmune manifestations, with autoimmune thrombocytopenia (AITP) being one of the most frequently observed hematological complications in affected individuals (3–6). This dual burden poses a unique clinical challenge in managing CVID patients presenting with AITP.

The management of AITP in patients with CVID requires a multifaceted approach that addresses both the autoimmune component and the underlying immunodeficiency. Historically, high-dose intravenous immunoglobulin (IVIG) therapy has been considered as the gold standard for managing CVID and AITP, providing exogenous immunoglobulins to sustain the immune system and suppress autoimmune responses (2, 7, 8). In recent years, subcutaneous immunoglobulin (SCIG) has emerged as a viable alternative to IVIG. SCIG offers the advantages of home-based administration and fewer systemic side effects, providing patients with a more flexible treatment option (9–11). The purpose of this study was to address this clinical challenge by prospectively evaluating the safety and efficacy of subcutaneous immunoglobulin (SCIG) in comparison to conventional IVIG treatment for the prevention of cytopenias in patients with both CVID and AITP.

## Methods

In this prospective observational study, we enrolled 55 patients who had previously participated in a retrospective observational study on the same topic (12). Individuals had a confirmed diagnosis of Common Variable Immunodeficiency (CVID) and a history of at least one thrombocytopenic event at some point in their medical history. The CVID diagnosis was based on the diagnostic criteria established by the European Society for Immunodeficiencies (ESID) (13, 14).

Among the 55 participants, 38 were recruited at the Center for Chronic Immunodeficiency, University Medical Center Freiburg, Germany, while the remaining 17 were from the Royal Free Hospital in London, UK. During the 5-year study, five patients were lost to follow-up, two patients died due to Hodgkin’s Lymphoma, and one due to autoimmune hemolytic anemia, respectively.

Hence, our analysis focused on data collected from 47 patients over 64 months, between July 2017 and April 2022. To investigate the incidence of thrombocytopenic events, we collected information regarding clinical manifestations, laboratory results, WHO Bleeding Scale (15), immunological findings, and immunoglobulin replacement therapy.

The primary endpoint of our study was the occurrence of severe thrombocytopenic events, defined as a platelet count of less than 50,000/µl in the presence of bleeding episodes or less than 20,000/µl regardless of bleeding. We evaluated the safety and efficacy of subcutaneous immunoglobulins (SCIG) in comparison to intravenous immunoglobulins (IVIG) for preventing autoimmune thrombocytopenia (AITP). Efficacy was determined by closely monitoring changes in platelet counts and the occurrence of bleeding events over the 64-month observation period. We calculated the incidence of thrombocytopenic events by summing occurrences within each 6-month interval when patients had documented thrombocytopenia. This calculation considered both routine blood tests, typically conducted every 6 months, and any additional tests performed during episodes of thrombocytopenia. Simultaneously, we determined safety by documenting any adverse events associated with the administration of SCIG or IVIG. Adverse events were defined as any unexpected medical occurrence associated with the immunoglobulin infusion, and they were categorized based on their nature and severity. Adverse events were systematically recorded, and any serious events were thoroughly investigated and reported following regulatory requirements and ethical guidelines.

Statistical analysis was conducted using R (R Foundation for Statistical Computing, Vienna, Austria; www.R-project.org) version 4.3.1, with statistical significance defined as p <0.05.

Continuous variables are reported as means with standard deviations or medians with interquartile ranges, while categorical variables are presented as frequencies and percentages.

Comparative analyses between the SCIG and IVIG groups were performed using the non-parametric Chi-square test and Mann–Whitney *U*-test. Results are illustrated through box plot diagrams, with boxes representing the lower quartile, the median, and the upper quartile, while the whiskers show the 10th and 90th percentiles. The occurrence of primary study endpoint was assessed by the Kaplan-Meier method and log-rank test. We applied Cox proportional hazards regression to calculate hazard ratios (HR) of time-to-event. Based on previous studies (12, 16, 17), the following covariates were evaluated as potential predictors associated with AITP.(1) Type of immunoglobulin substitution, (2) immunomodulatory treatment, (3) spleen size, and (4) IgG trough levels. The analysis was performed by the *survival* and *survminer* packages in Rstudio.

All participants provided written informed consent for the study. This study was conducted in accordance to the ethical guidelines outlined in the Declaration of Helsinki and was approved by the institutional review boards of both centers. Ethics protocol numbers: No. 295/13 for the University Medical Center Freiburg, Freiburg, Germany, and No. 04/Q0501/119 for the Royal Free Hospital, University College London, Institute of Immunity and Transplantation, London, UK.

## Results

### Study participants

We performed our final analysis on a cohort of 47 patients. Demographic and clinical characteristics of the cohort are shown in **Table 1**. Thirty-five patients were enrolled at the Center for Chronic Immunodeficiency, University Medical Center Freiburg, Germany, and 12 patients were recruited at the Royal Free Hospital, London, UK. Twenty patients (43%) were on intravenous immunoglobulin (IVIG) replacement and 27 patients (57%) received immunoglobulin through the subcutaneous route (SCIG). No patients were transitioned between IVIG and SCIG therapies during this study.

**Table 1:** Characteristics of study population.

ID	Gender	Age (years)	IgG substitution	Dose (mg/month)	Spleen status	SARS-CoV2 infection	AITP bout	Long-term immunomodulation	Other manifestations
1	Female	56	SC	48000	splenomegaly	yes	no	no	AIHA
2	Female	43	IV	40000	normal	no	no	no	no
3	Male	61	IV	40000	splenomegaly	no	yes	steroids	GLILD
4	Male	60	SC	48000	splenomegaly	no	no	no	no
5	Female	60	SC	48000	normal	no	no	no	no
6	Male	47	IV	48000	splenectomy	yes	no	no	no
7	Male	51	SC	48000	normal	no	no	steroids	GLILD
8	Male	61	SC	48000	normal	no	no	steroids	GLILD
9	Female	63	IV	32000	splenomegaly	no	no	steroids	GLILD
10	Male	67	SC	32000	normal	no	no	no	no
11	Female	59	SC	32000	normal	no	no	no	no
12	Male	52	SC	40000	normal	no	no	no	no
13	Female	47	SC	32000	normal	yes	no	no	no
14	Female	38	SC	40000	normal	no	no	no	AIN
15	Female	59	IV	80000	splenomegaly	no	no	Steroids, Upadacitinib	rheumatoid Arthritis
16	Male	51	SC	40000	splenomegaly	no	no	steroids	GLILD
17	Male	35	IV	60000	splenomegaly	no	no	steroids	GLILD
18	Male	54	SC	64000	splenomegaly	no	yes	Abatacept	GLILD
19	Female	72	IV	32000	splenomegaly	no	yes	no	no
20	Male	63	IV	30000	normal	yes	no	no	no
21	Male	49	SC	36000	normal	no	yes	Cyclosporin	Enteropathy
22	Male	30	SC	48000	normal	no	no	no	no
23	Male	44	SC	36000	normal	no	no	no	no
24	Male	31	SC	48000	normal	no	no	Abatacept	Enteropathy
25	Male	40	IV	30000	splenectomy	no	no	no	no
26	Male	70	SC	32000	normal	no	no	no	Enteropathy
27	Female	52	SC	32000	normal	no	no	steroids	GLILD
28	Female	64	IV	25000	normal	no	no	no	no
29	Female	48	SC	40000	splenomegaly	no	yes	no	no
30	Female	43	IV	45000	splenomegaly	no	yes	no	no
31	Male	36	SC	32000	normal	no	yes	no	no
32	Female	51	SC	40000	normal	no	no	no	no
33	Female	57	IV	40000	normal	no	yes	steroids	GLILD
34	Female	58	SC	32000	normal	no	yes	no	no
35	Female	44	SC	24000	normal	no	yes	no	no
36	Female	46	SC	40000	splenomegaly	NA	no	no	no
37	Female	67	SC	30000	splenectomy	NA	no	steroids	GLILD
38	Female	46	IV	25000	splenomegaly	NA	no	no	no
39	Female	72	IV	40000	normal	NA	yes	steroids	Enteropathy
40	Female	60	IV	40000	splenomegaly	NA	no	mycophenolate mofetil	GLILD
41	Female	61	IV	35000	splenomegaly	NA	no	no	no
42	Female	50	IV	35000	splenomegaly	NA	no	no	no
43	Female	41	SC	40000	splenomegaly	NA	yes	no	no
44	Female	45	SC	40000	splenectomy	NA	no	mycophenolate mofetil	GLILD
45	Female	43	IV	35000	splenomegaly	NA	no	steroids	GLILD
46	Female	54	IV	35000	splenomegaly	NA	no	no	no
47	Female	58	IV	40000	splenomegaly	NA	no	steroids	GLILD

SC, subcutaneous; IV, intravenous; AITP, autoimmune thrombocytopenia; AIHA, autoimmune haemolytic anemia; GLILD, Granulomatous Lymphocytic Interstitial Lung Disease; AIN, autoimmune neutropenia.

Every patient maintained a consistent IgG replacement dosage. The median dosage was 577.2 mg/kg/month (range: 271–869 mg/kg/month), and the target trough level was IgG ≥7 g/l. Participants had platelet count and IgG trough level determined every 6 months, and additionally during episodes of thrombocytopenia. On average, patients on IVIG substitution had infusions scheduled every four weeks. SCIG patients had their infusions scheduled as follows: twenty-three patients had one injection per week and four patients had two injections per week.

No drug-related adverse events in causal relation to the administration of SCIG or IVIG were reported throughout the observation period among the enrolled participants during the study.

### Treatment and course of AITP

During the observation period, twelve patients (25,5%) had an AITP bout (**Table 1**). Seven patients were on IVIG replacement and five were on SCIG. The WHO Bleeding Scale was employed to categorize the severity of bleeding symptoms at the initial diagnosis. In our cohort, the majority of patients experienced only mild bleeding symptoms: Grade 0 (n=4, 33.3%), Grade 1 (n=7, 58.3%), and Grade 2 (n=1, 8.3%). Three patients experienced at least one relapse during the observation period following the registered retrospective AITP event. For the treatment of the AITP bouts, all patients received corticosteroids as first-line treatment. Two patients required a second-line treatment, which included rituximab (n=1) and fostamatinib (n=1).

### Autoimmunity and spleen size

From the total cohort, spleen size was reported as normal in 23 patients (48.9%), while splenomegaly was reported in 20 patients (42.5%). Additionally, splenectomy was documented in four patients (8.5%). These splenectomies were performed prior to the observation period in 1995, 1999, 2006, and 2008. Three of the splenectomized patients had treatment-resistant AITP, while one underwent splenectomy due to treatment-resistant autoimmune hemolytic anemia (AIHA) and autoimmune neutropenia (AIN).

Among the 12 patients who experienced an AITP episode, other cytopenic events were observed in one patient with AIHA and another with autoimmune neutropenia. Notably, these events did not occur simultaneously with AITP bouts and were managed with steroids only, or with steroids and granulocyte-colony-stimulating factor (G-CSF), respectively. Fifty percent of these 12 patients (n=6) had splenomegaly.

We compared the duration of immunoglobulin therapy since diagnosis between patients with and without splenomegaly, and between those with and without autoimmunity, using the Mann-Whitney U test. The mean therapy duration did not differ significantly between patients with (23.05 + 6.81 years) and without splenomegaly (21.29 + 4.60 years; p = 0.680). Similarly, patients with autoimmunity had a mean duration of 20.41 + 4.01 years compared with 22.60 + 6.06 years in those without autoimmunity (p = 0.084). Thus, in our cohort, neither splenomegaly nor autoimmunity was associated with differences in the duration of immunoglobulin therapy since diagnosis.

### Immunosuppressive therapy

Nineteen patients had immunosuppressive treatment for conditions different than cytopenia. Fourteen patients (n=14, 29.7%) had granulomatous–lymphocytic interstitial lung disease (GLILD). Among these patients, 11 received steroids, one received abatacept, and two received mycophenolate mofetil. Enteropathy was documented in four patients (n=4, 8.5%), one treated with a gluten-free diet, one with steroids, one with abatacept, and one with cyclosporine. Additionally, one patient (n=1, 2.1%) was diagnosed with seronegative rheumatoid arthritis and received upadacitinib and steroids as treatment.

### Primary outcome

**Figure 1:**
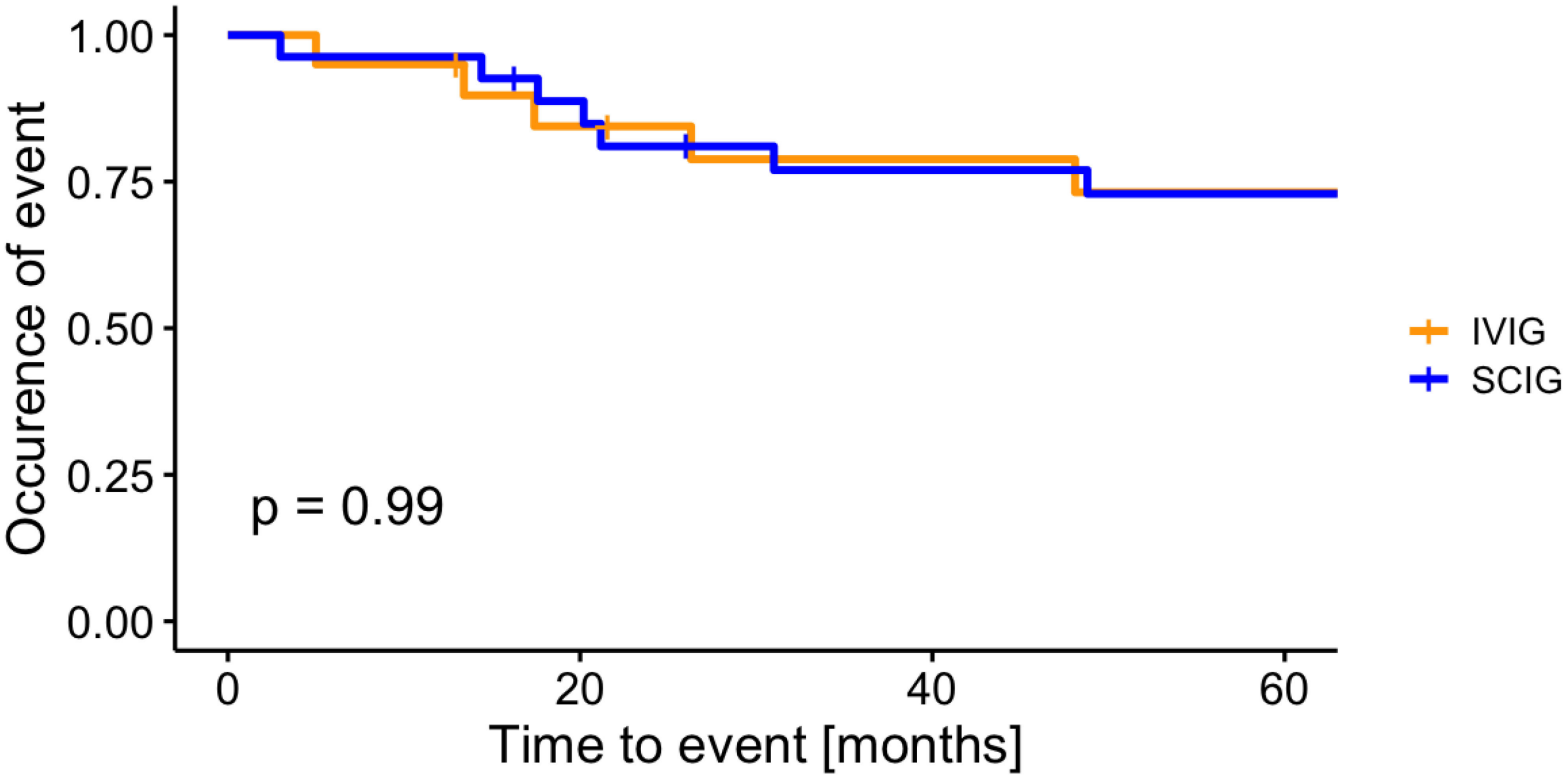
No difference in the occurrence of AITP between patients in the IVIG-group and the SCIG-group. Kaplan-Meier survival curves illustrating the incidence of thrombocytopenic events in patients treated with subcutaneous immunoglobulin (SCIG) and intravenous immunoglobulin (IVIG). Group comparisons, assessed via the log-rank test, reveal no statistically significant differences between the two treatment modalities (p = 0.99).

**Figure 2:**
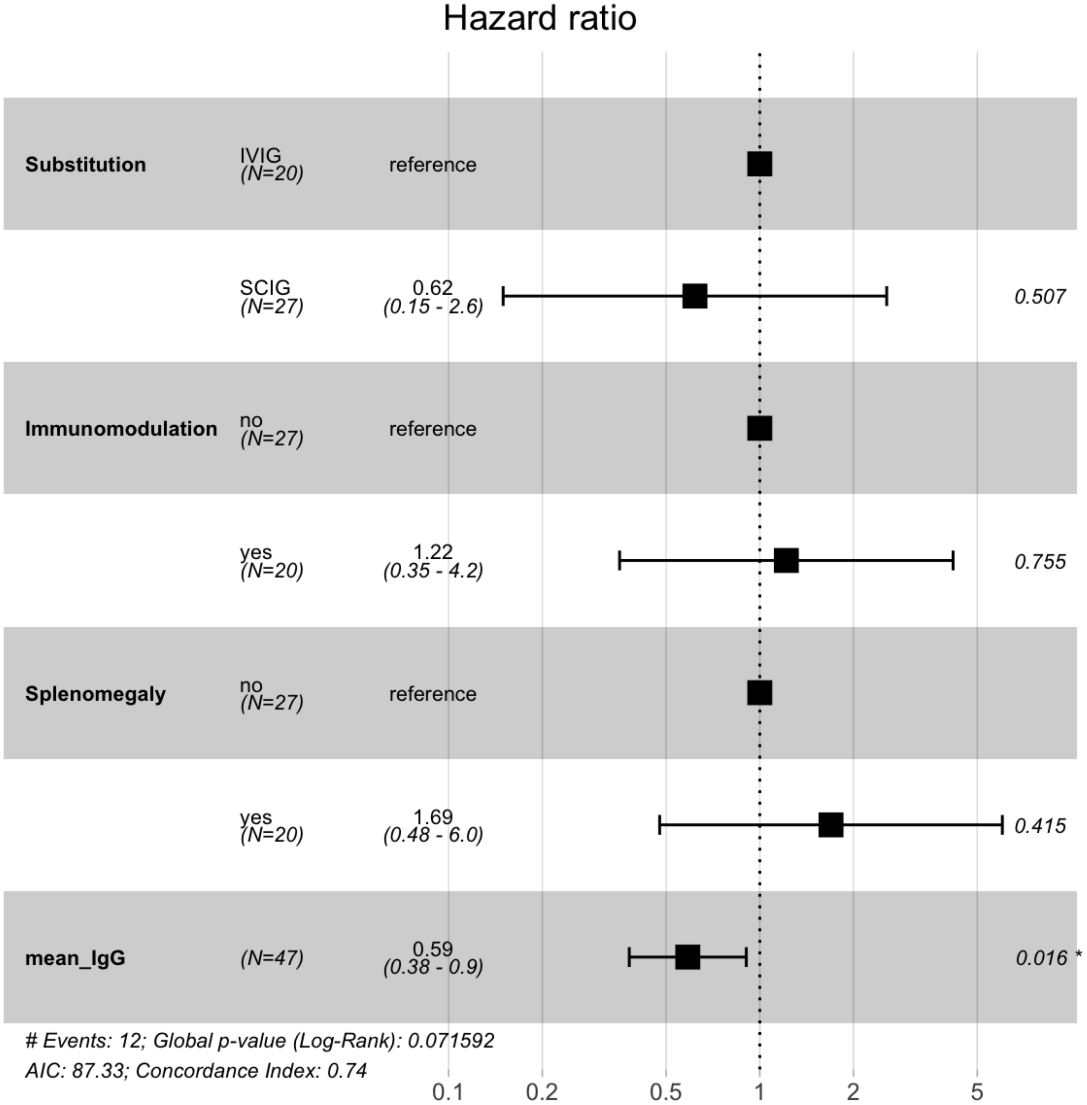
Low IgG trough levels are a key factor for the development of thrombocytopenic events. The figure highlights the significance of low IgG levels as a pivotal factor in the development of thrombocytopenic events. The forest plot represents the hazard ratio (HR) and the corresponding 95% confidence intervals for each covariate considered in the Cox proportional hazards model. Magnitude of significance is denoted with asterisks (*).

Compared with individuals receiving IVIG, patients under SCIG substitution did not show significant differences in the occurrence of thrombocytopenia (p = 0.99) in the unadjusted Kaplan-Meier analysis (**Figure 1**). We further examined the time-to-event between the two groups adjusting for other factors associated with AITP (**Figure 2**). Patients receiving IVIG did not show differences when compared with patients receiving SCIG in the occurrence of AITP with an HR 0.62 (95% CI, 0.15-2.6; p=0.507). No differences were observed for spleen status with an HR 1.69 (95% CI, 0.48-6.0; p=0.415), and for immunomodulatory treatment with an HR 1.22 (95% CI, 0.35-4.2, p=0.755). However, individuals with higher IgG trough levels had a lower risk for a thrombocytopenic event HR 0.59 (95% CI, 0.38-0.9, p=0.016).

**Figure 3:**
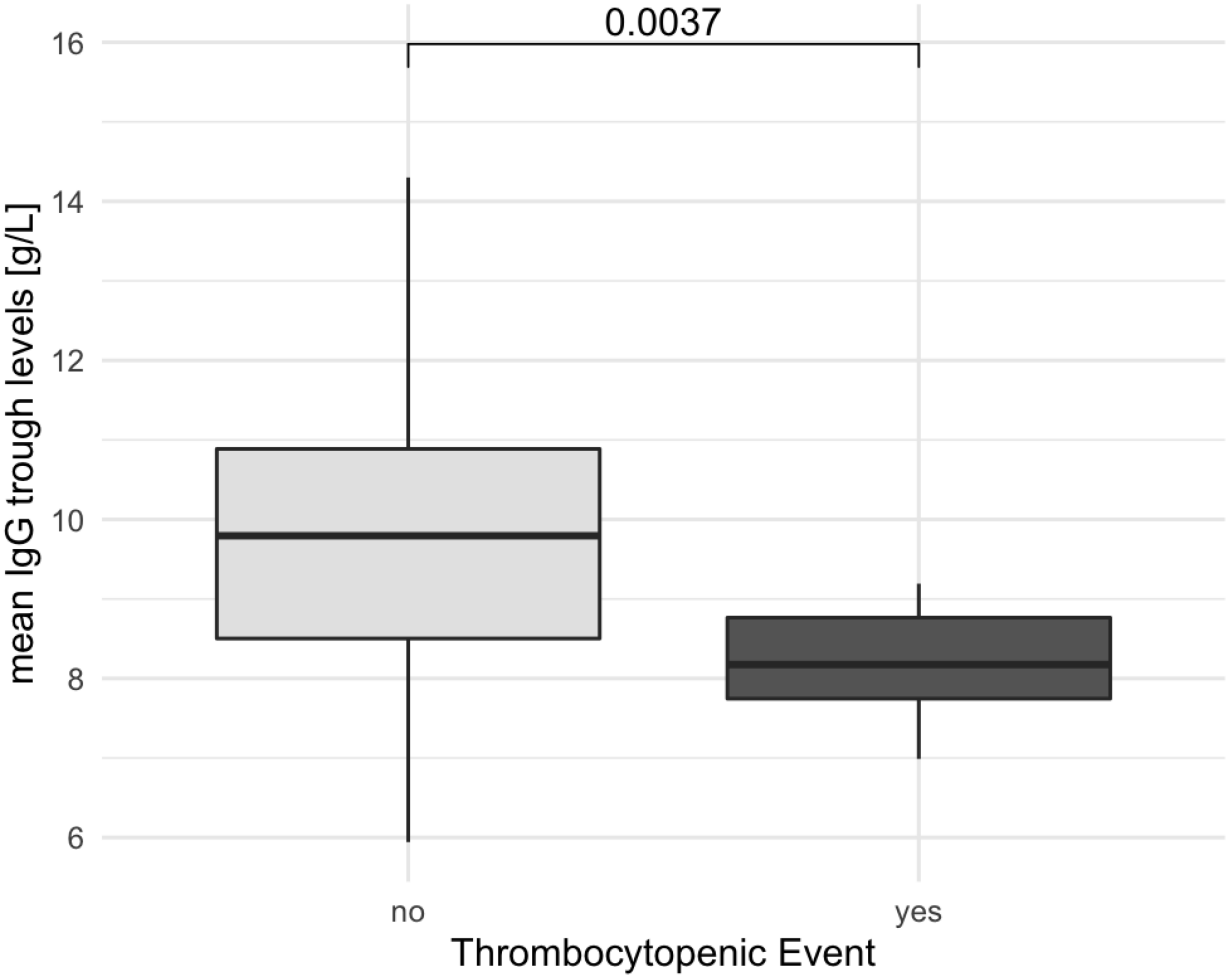
Patients with thrombocytopenic events show lower IgG trough levels. The figure depicts the association between patients experiencing thrombocytopenic events and lower IgG levels. Box plot diagrams represent the lower quartile, the median, and the upper quartile, while the whiskers show the 10th and 90th percentiles. Differences were compared by the Mann–Whitney U-test. p is considered significant when < 0.05.

**Figure 4:**
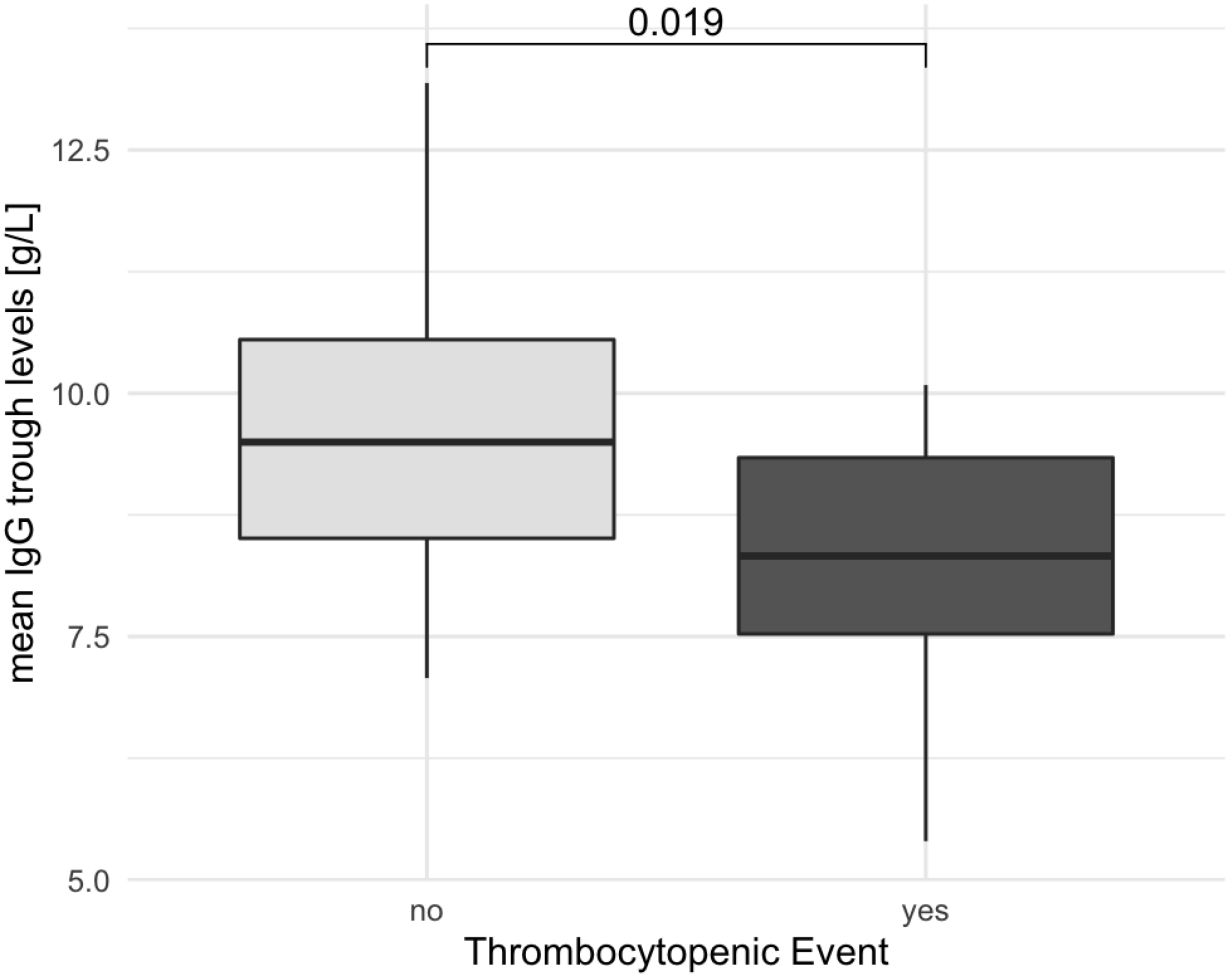
Association between thrombocytopenic events and lower IgG levels in CVID patients over 10 years. The figure depicts the association between patients experiencing thrombocytopenic events and lower IgG levels combining data from both retrospective and prospective cohorts. The statistically significant difference in IgG trough levels between AITP and non-AITP individuals is underscored by the Mann–Whitney U-test results (p= 0.019). Box plot diagrams represent the lower quartile, the median, and the upper quartile, while the whiskers show the 10th and 90th percentiles. p is considered significant when < 0.05.

We also examined the IgG trough levels between patients with an AITP bout compared to the non-AITP group (**Figure 3**). Trough levels used for the analysis represent the average level among measurements. Patients who were affected by AITP had a lower IgG trough level (mean AITP 8.23 ± 1.16 g/L, mean non-AITP 9.74 ± 2.06 g/L, p=0.0037). Expanding our analysis, we combined data from both the retrospective and prospective cohorts, as illustrated in **Figure 4**. Once again, individuals with AITP exhibited significantly lower IgG trough levels (mean 8.26 ± 1.44 g/L) in comparison to the non-AITP group (mean 9.35 ± 1.72 g/L, p= 0.019).

### SARS-CoV2 infection

**Figure 5:**
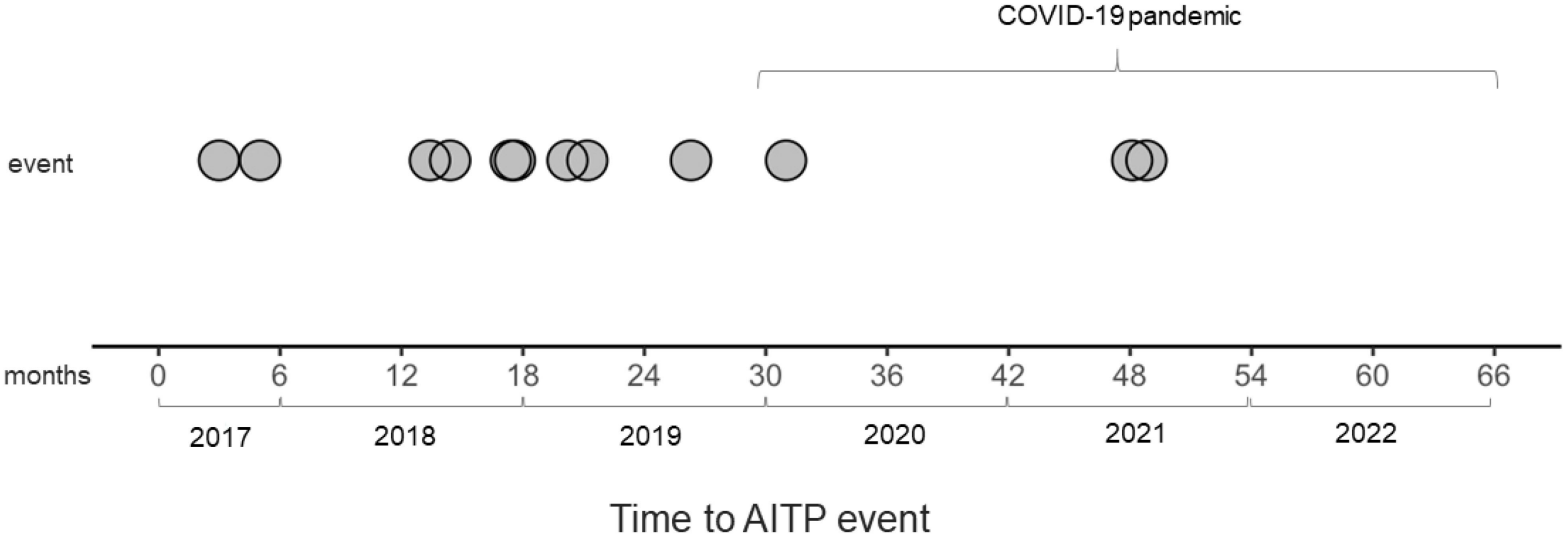
Impact of the pandemic on the occurrence of Autoimmune Thrombocytopenic Purpura (AITP) events. The plot shows no discernible increase in the number of AITP events during the COVID-19 pandemic. Time-series plot illustrating the occurrence of AITP events throughout the observation period. Each circle on the graph represents an individual AITP event.

None of the patients presented an AITP bout related to a SARS-CoV2 infection. During the observation period, six patients had a documented SARS-CoV2 infection. Five had upper respiratory symptoms with a disease duration of 13–82 days, but one was hospitalized due to pneumonia and the infection lasted for 22 days. It is noteworthy that the number of AITP events did not increase during the pandemic (**Figure 5**).

## Discussion

In our previous retrospective study (12), we observed that the route of immunoglobulin replacement therapy had no evident impact on the occurrence of AITP. These initial findings motivated us to do a five-year follow-up of the same cohort, aiming to improve the clinical characterization of patients.

Our extended prospective study confirmed the results from our retrospective study, showing that subcutaneous and intravenous IgG replacement therapies do not differ in their protection towards AIHA, as there was no significant difference in the occurrence of AITP. Nevertheless, when we adjusted for other influential factors associated with thrombocytopenia, a significant pattern emerged: Patients with lower IgG trough levels exhibited a higher risk of AITP when compared with those with higher IgG trough levels. This supports our previous study, wherein we had identified that an IgG level below 7g/L was associated with an increased risk of AITP (12). The combined analysis of data from the retrospective and prospective cohorts over a 10-year period strengthens the notion that low IgG trough levels are a risk factor for autoimmune cytopenias in CVID patients. Regarding SARS-CoV-2 infection, there was no significant increase in the number of AITP events during the pandemic. A possible explanation is the reduced exposure to infectious triggers.

The primary goal of immunoglobulin replacement therapy is the prevention of infections. Previous studies have demonstrated the effectiveness of IgG replacement in preventing autoimmune cytopenias in patients with CVID and concurrent AITP. These studies not only revealed positive responses to low-dose immunoglobulins but also the potential to reduce the use of steroid treatments (18, 19). Additionally, research conducted by Somasundaram et al. (20) claims that in patients with CVID-related AITP, the clinical course of AITP tends to be mild. In line with these findings, the majority of patients in our cohort responded in case of AITP bouts to high-dose steroid treatments. Only two individuals required second-line therapies.

This study has some limitations. Its statistical power is constrained, and the two-center study design may have introduced an inherent bias. Our primary focus was mitigating type I errors while analyzing group differences through statistical tests. However, the relatively modest sample size raises the potential for type II errors, which were not accounted for in our analyses.

In summary, our findings indicate that CVID patients receiving SCIG did not show a higher incidence of thrombocytopenic events compared to those receiving IVIG. Moreover, our results point to a low IgG trough level as a risk factor for the development of AITP. This emphasizes the importance of monitoring trough levels to ensure they are appropriate (i.e. > 7 g/L). We hypothesize that in patients on SCIG with higher IgG trough levels (i.e. > 7 g/L), immunoglobulin replacement therapy may also exert an immunomodulatory effect controlling autoimmune (thrombo)cytopenic events.

## Data Availability

The original contributions presented in the study are included in the article/supplementary material. Further inquiries can be directed to the corresponding author.
